# Interleukin-13 Receptor Alpha 2-Targeted Glioblastoma Immunotherapy

**DOI:** 10.1155/2014/952128

**Published:** 2014-08-27

**Authors:** Sadhak Sengupta, Bart Thaci, Andrew C. Crawford, Prakash Sampath

**Affiliations:** ^1^Roger Williams Medical Center Brain Tumor Laboratory, 825 Chalkstone Avenue, Prior 222, Providence, RI 02908, USA; ^2^Department of Neurosurgery, Boston University School of Medicine, Boston, MA 02118, USA; ^3^University of Illinois College of Medicine, Urbana-Champaign, IL 61801, USA

## Abstract

Glioblastoma (GBM) is the most lethal primary brain tumor, and despite several refinements in its multimodal management, generally has very poor prognosis. Targeted immunotherapy is an emerging field of research that shows great promise in the treatment of GBM. One of the most extensively studied targets is the interleukin-13 receptor alpha chain variant 2 (IL13R*α*2). Its selective expression on GBM, discovered almost two decades ago, has been a target for therapy ever since. Immunotherapeutic strategies have been developed targeting IL13R*α*2, including monoclonal antibodies as well as cell-based strategies such as IL13R*α*2-pulsed dendritic cells and IL13R*α*2-targeted chimeric antigen receptor-expressing T cells. Advanced therapeutic development has led to the completion of several clinical trials with promising outcomes. In this review, we will discuss the recent advances in the IL13R*α*2-targeted immunotherapy and evaluate the most promising strategy for targeted GBM immunotherapy.

## 1. Introduction

Despite incremental improvements in survival with the current standard of care for glioblastoma (GBM), which is a tripartite regimen of surgery, radiotherapy, and chemotherapy [1, 2], the prognosis for most patients remains dismal [3, 4]. Major limitations in the treatment of GBM are the tumor's location within the brain that impedes delivery of cytotoxic agents across the blood-brain barrier [2], compounded with a strong immunosuppressive environment [5] and chemo- and radioresistant glioma-initiating cells [6, 7]. As a result, novel strategies are continually being tested to improve patient survival, quality of life, and overall outcomes.

Targeted immunotherapy has thus emerged as promising field of research in the treatment of malignancies and has received a great deal of interest in recent years [8, 9]. Optimism regarding the use of targeted immunotherapy has been even higher recently, since the reported cure of lymphoma patients with engineered or genetically modified T cells targeting CD19 malignant cells [10]. This has increased the focus towards the potential antigens present exclusively in glioma as targets for gene- and immunotherapy. One of the most extensively studied targets is the interleukin-13 receptor alpha 2 (IL13R*α*2) [11]. IL13R*α*2 is a decoy receptor for interleukin-13 (IL13), lacking the signaling chain that is present on the ubiquitous IL13R*α*1, thus preventing any IL13-mediated downstream signaling pathway [12]. Further, higher affinity of IL13 to IL13R*α*2 allows for sequestration of the ligand away from IL13R*α*1. Increased expression of IL13R*α*2 has been reported to promote tumor progression in glioma and other tumor models. IL13R*α*2 expression is a prognostic marker for glioma malignancy grade and for poor patient survival [13]. Its selective expression on MG, discovered almost two decades ago, has been a target for therapy ever since [14]. Several targeted therapies have been developed against IL13R*α*2 on MG including bacterial toxins conjugated to IL13 [15], nanoparticles [16, 17], oncolytic virus [18, 19], as well as immunotherapies using monoclonal antibodies [20], IL13R*α*2-pulsed dendritic cells [21], and IL13R*α*2-targeted chimeric antigen receptors [22, 23]. Advanced therapeutic development has led to the completion of phase I clinical trials for IL13R*α*2-targeted chimeric antigen receptors and phase III clinical trials for bacterial toxins [11]. Here we will review the immunotherapeutic modalities that have been developed to specifically target IL13R*α*2-expressing GBMs.

## 2. Using Monoclonal Antibodies to Target IL13R***α***2 on GBMs

Both hybridoma technology or phage display libraries have been used extensively to generate monoclonal antibodies against IL13R*α*2 [20, 24]. It is to be noted that antibodies generated by phage display technology tend to have a lower affinity of binding to the target than hybridoma generated antibodies. Balyasnikova et al. showed that IL13R*α*2-targeting monoclonal antibody generated by hybridoma technology exhibited high affinity towards glioma cells, both* in vitro* and* ex vivo* [20]. This is also the only study that found that the high affinity antibody also prolonged survival in mice when coinjected intracranially with glioma cells. Another clear path indicated by the high specificity targeting of this study is the possibility of delivering antibodies systemically with fewer side effects. Studies have shown that intraperitoneal or intravenous delivery of antibody fragments may successfully home to glioma and reduce its growth in flank or orthotopic models [24]. More work is needed on this approach to determine its potency. One must be wary of trading potency for specificity, as targeting very specific amino acid sequences in highly mutated tumors may result in killing only a subgroup of cells. Also, the advantage of increased specificity via antibody based targeting must be weighed against decreased potency as compared to the IL13 ligand approach. Kioi et al. found that none of the IL13R*α*2 antibody fragment variants conjugated to pseudomonas exotoxin (PE) could match the potency of IL13-PE fusion chimera (IL13PE38QQR)* in vitro* or* in vivo*. Keeping that in mind, a better approach might be to generate immune responses towards a variety of specific glioma antigens.

## 3. Dendritic Cells Pulsed with Tumor-Associated Antigens

One of the strongest immune-evasion techniques employed by GBM is poor antigen presentation by the tumor cells, and most patients with high-grade gliomas have very weak systemic response to the tumor antigens [25]. Dendritic cell based immunotherapy has been used extensively to counteract the GBM immune-evasion characteristics. Recent approaches include* ex vivo* pulsation of dendritic cells with glioma antigens, where cells of interest were sorted from GBM patient's peripheral blood mononuclear cells and exposed to glioma-associated tumor antigens in presence of immunostimulatory cytokines. The loaded cells were then injected back into the respective patients to observe the increased immune response. One must be critical in this approach during the choice of antigens used to stimulate the dendritic cells. Instead of exposing the cells to lysates, which offer complex cocktail of different antigens, more targeted immune response can be affected by pulsation of the dendritic cells with purified tumor-associated peptides such as IL13R*α*2, EGFRvIII, or gp100. Dendritic cells pulsed with GBM antigens are being tested in phase I/II clinical trials. Robust immune response has been observed in a subgroup of patients with HLA-A*24/A*02 allele when they were injected with dendritic cells pulsed only with IL13R*α*2 peptides [26, 27]. In a recent study with ICT-107, an intradermally administered autologous vaccine, from dendritic cells pulsed with several different antigens including IL13R*α*2, showed a statistically significant increase in progression-free survival (NCT01280552). Median progression-free survival increased by 2 months overall. A group of patients that received at least 4 induction vaccinations showed an even longer median progression-free survival [28]. IL13R*α*2 peptides have also been part of different cocktails of immunogenic molecules to provide more extensive coverage to different cell populations [21, 29]. Promising results have been observed in some of these antigen-cocktail pulsed dendritic cells being used in clinical trials. The benefits of IL13R*α*2 based vaccines are manifold as they are not limited to GBM. Subcutaneous vaccinations with synthetic peptides for tumor-antigen epitopes that include IL13R*α*2, WT1, survivin, and EphA2 in a recent study by Okada et al. showed low toxicity and potent immune response in low-grade glioma [30].

## 4. Chimeric Antigen Receptor-Modified T Lymphocytes

Genetic manipulation of autologous T cells to specifically target a particular tumor antigen is a novel and alternative strategy to bypass the failure of cytotoxic immune response induction by most tumor cells [31–33]. The application of chimeric antigen receptors (CAR) for immunogene therapy of malignant tumors is a promising strategy in which an antibody or ligand binding domain is fused with the zeta signaling chain of the T cell receptor [32–35]. The resulting CAR T cells are redirected by the neospecificity to attack tumors expressing the surface antigen or receptors recognized by the gene-modified T cell receptors and provide cellular therapy that attacks the tumor through normal host immune response in a highly regulated fashion. These cells are free to roam throughout the brain and systemic circulation, making the need for colocalization and bioavailability less of a problem.

The first generation CAR T cells had a single chain variable fragment (scFv) of an antibody specific to the target or a targeting ligand (such as IL13) connected with intracellular signaling zeta-domain of CD3 (CD3*ζ*). Weak activation levels in these CAR T cells were resolved with the addition of a CD28 costimulatory domain in the second-generation CARs, which enhanced proliferation, cell survival, and memory formation as well as significantly increased cytotoxicity [36].

First- and second-generation CAR T cells have been successfully used in targeting IL13R*α*2. These CARs were designed by modifying the IL13 molecule by site-directed mutagenesis to change its affinity to IL13R*α*2. Kahlon et al.designed a CAR with an IL13 mutein with a mutation at the E13 site (IL3.E13Y zetakine) [22], while another IL13CAR by Kong et al. was designed to have two mutations, one at site E13 and another at R109, in IL13 molecule (IL13.E13 K.R109 K) [23]. The double mutation allowed for increased specificity towards IL13R*α*2 and decreased affinity towards IL13R*α*1. All the studies conducted so far have relied on local intracranial injections to establish efficacy. More studies need to be done to establish risk levels for adverse effects.  Table 1 shows the different CARs developed to target IL13R*α*2-expressing GBMs.

### 4.1. IL3 Zetakine

It has been established through early successful preclinical studies on first-generation CAR T cells that the IL13.E13Y zetakine (IL13 zetakine) T cells induced secretion of IFN*γ*, TNF*α*, and GM-CSF only in the presence of IL13R*α*2-expressing tumor cells. When cultured* in vitro* together with glioma cells, they also lysed only IL13R*α*2-expressing U251 glioma cells [22]. Immunodeficient mice, which were given intratumoral injection of IL13 zetakine T cells, were cured of intracranial glioma and also did not show tumor recurrence. The lack of recurrence was predicted to be due to the lack of resistance towards such therapy in glioma. One drawback of most other currently tested therapies is tumor recurrence, as a certain group of cells that is resistant to the therapy becomes the dominant phenotype or acts as a cell pool to originate tumor recurrence. This group of cells, referred to as cancer stem cells or in case of GBMs as glioma-initiating cells, often expresses the classical markers of stem cells, is self-renewing, and can initiate tumor formation* in vivo*. Like many other solid tumors, glioma-initiating cells have been shown to be responsible for resistance to current therapies and for tumor recurrence. However, the glioma-initiating cells derived from IL13R*α*2+ tumors express IL13*α*R2 at levels similar to differentiated cells and were similarly sensitive to* in vitro* IL13 zetakine therapy [41].

The potential for targeting IL13R*α*2-expressing GBMs has been demonstrated by early clinical experience at City of Hope in two phase I clinical trials with intracranial administration of first-generation IL13 zetakine T cell clones in patients with high-grade gliomas. In the pilot trial (NCT00730613), 3 consented participants with recurrent/refractory GBM were treated with autologous first-generation IL13 zetakine T-cell clones in escalating cell dose infusion cycles up to 10^8^ [37]. In order to facilitate the study, the CAR T cells also expressed a hygromycin resistance gene/herpes simplex virus 1 thymidine kinase fusion (HyTk) and a PET reporter gene. A case study on one of the research participants has been reported with respect to the noninvasive detection of the autologous IL13 zetakine/HyTk+ T cells using 18F-FHGB PET postadoptive transfer [42]. CAR T cells were detected at the site of injection, as well as at a secondary site of recurrence near the corpus callosum, providing evidence for detection of CAR T cells and suggesting the potential of CAR T cells for trafficking to the sites of infiltrative diseases. In the second phase I clinical trial (NCT01082926) involving 6 research participants, an allogenic CAR CD8+ T cell, termed GRm13Z40-2, generated from a healthy donor was modified to express the first generation IL13 zetakine/HyTk CAR as described above [38]. These T cells also had their glucocorticoid receptor sites deleted to make the T cells resistant to steroids following adoptive transfer. All 6 patients had nonresectable recurrent/refractory GBMs. They were treated in conjunction with IL2 with repetitive doses of 10^8^ CAR T cells. In all the participants in both clinical trials, the feasibility of this approach was demonstrated clinically, with minimal therapy related side effects and provided evidence for transient antiglioma responses for patients with IL13R*α*2-expressing tumors [11].

### 4.2. IL13 CAR

In a recent publication by Kong et al. [23], a second-generation IL13CAR composed of a mutant IL13 (IL13.E13K.R109K) extracellular domain linked to intracellular signaling elements of the CD28 costimulatory molecule and CD3*ζ* was reported. In comparison to the Il13.E13Y zetakine, which was designed to be delivered via direct transfection of the CAR-coding plasmids, this IL13CAR was delivered to the T cells by retrovirus, which increased the transduction efficiency to as high as 79%. The double mutant IL13 vastly improved specificity against IL13R*α*2+ tumors while showing little affinity for IL13R*α*1 expressing cells. IL13R*α*2+ glioma targets were accurately targeted and eliminated by the CAR expressing T cells with abundant secretion of cytokines IL2 and IFN*γ*. Marked increase in animal survival was observed in an* in vivo* test with a human glioma xenograft model with a single intracranial injection of CAR expressing designer T cells into tumor sites.

### 4.3. HER2.CD28 and IL13R*α*2.CD28 biCAR T Cells

Immune escape is often associated with targeted immunotherapy of GBMs due to antigen heterogeneity or unavailability of receptor sites on the surface of solid tumors. Tumor cells also employ immune-evasion techniques to escape immune recognition [5]. GBMs notorious for their antigenic heterogeneity often express varied antigen profile within single tumors and between patients [43, 44]. Hegde et al. performed a tandem expression of anti-HER2 and anti-IL13R*α*2 CARs in single T cells and showed that combinational targeting with the bispecific CAR T cells, in comparison to unispecific CAR T cells (anti-HER2 and anti-IL13R*α*2 resp.), was able to offset antigen escape and enhanced effector activity against GBM patient tumor cells as well as xenograft murine glioma model [39].

## 5. Effects of Steroids and Chemotherapy on GBM Immunotherapy

Currently, the most important form of medical treatment for GBM is surgical resection. The success of this surgical intervention is based on perioperative management of the patient. Steroids are commonly used preoperatively to reduce the symptoms of mass effect and edema caused by the tumor [45]. The timing and dose of steroids varies according to surgeon preference. A common regimen for adults is dexamethasone, 6 mg intravenously or orally every 6 hours. If mass effect is profound, doses as high as 20 mg every 4 hours may be considered [45]. Recent studies have shown that dexamethasone reduces tumor-induced disturbances of the microenvironment such as neuronal cell death and tumor-induced angiogenesis, inhibits glioma cell growth in a concentration and species-dependent manner, and executes neuroprotective effects [46–48]. However, surgical removal of the tumor is not curative and must be supplemented with additional therapies to prolong survival and reduce recurrence. Treatment with corticosteroids presents a number of challenges to current immunotherapeutic approaches. One major problem is that the administration of dexamethasone suppresses the immune system by reducing the proliferation of T cells [49]. There is a physical and functional interaction between the glucocorticoid receptor and the T cell receptor (TCR) complex. In its unligated state, the glucocorticoid receptor has an important role in TCR signaling, but, after glucocorticoid-receptor-ligand binding (caused by short-term treatment with the synthetic glucocorticoid dexamethasone), the TCR complex is disrupted, leading to impaired TCR signaling [50]. Dexamethasone acts to functionally suppress immune modulators, which result in fewer IFN-*γ*-producing Th1 cells and a greater number of IL-4-producing Th2 cells [51]. This becomes an issue for the administration of adoptive T cell therapy as well as the activation of other pathways. One study demonstrates the direct correlation between the use of steroids and the functionality of targeted T-cell immunotherapy. Treatment with D-CAR(+) T cells exhibited specificity for *β*-glucan which led to damage and inhibition of hyphal growth of* Aspergillus in vitro* and* in vivo*. Treatment of D-CAR(+) T cells with steroids did not compromise antifungal activity significantly [52]. Another problem involves corticosteroid-induced reduction in contrast enhancement on radiographic imaging, which has been seen with gliomas. This finding may represent a diagnostic dilemma. Concern that steroid-induced cytotoxicity obscures histological diagnosis of suspected lymphoma may lead to postponement of a biopsy. If glioma is not considered in the differential diagnosis, reduction in tumor contrast enhancement may be misinterpreted as disease regression rather than a transient radiographic change [53]. Treatment of GBM with corticosteroids has become a double-edge sword. Future studies should be directed towards finding an optimal balance between immune suppression and activation.

A limiting factor for GBM immunotherapy using adoptive cell therapy approach, like engineered T cells, is temozolomide- (TMZ-) induced lymphopenia. FDA-directed GBM standard care must include a tripartite therapy of surgical resection followed by radiation and TMZ chemotherapy, concurrently with radiation and then as an adjuvant [54]. TMZ is a DNA alkylating agent and is the most successful antiglioma drug that has added several months to the life expectancy of GBM patients [55]. TMZ on the other hand is also responsible for inducing lymphopenia and myelosuppression in malignant glioma patients undergoing chemotherapy [56–58]. Although TMZ-induced lymphopenia facilitates antitumor vaccination by inducing passive immune response, it has been also associated with poor immune surveillance leading to opportunistic infections in glioma patients [59]. Reduced expression of DNA-repair enzyme O-6-methylguanine-DNA-methyltransferase (MGMT) in mature monocytes [60, 61] and further deletion of MGMT by TMZ have been determined to be the cause of lymphopenia [58]. Still, the future of chemotherapy-resistant immunotherapy does not look much depressing. In recent developments, it has been shown that genetic modification of MGMT molecule has been shown to render chemoprotection against TMZ [62]. Recent studies have shown promising effects of chemoprotection in hematopoietic cells by mutating the proline residue at 140 of the MGMT peptide to lysine (P140KMGMT) [55]. A calculated approach of using similar chemoprotection during GBM-targeted adoptive T cell-mediated immunotherapy may facilitate concurrent chemotherapy and immunotherapy and thus help reduce therapy time.

## 6. Conclusion

Abundance of IL13R*α*2 overexpression in GBM is a well-documented fact [13, 14, 41, 63–65]. IL13R*α*2 is expressed in approximately 58% of adult and 83% of the pediatric brain tumors as well as on glioma-initiating cells [13, 41, 65]. This wealth of information has motivated the development of highly effective immunotherapies targeting IL13R*α*2 on GBMs as discussed in this review article (Table 2).

High specificity of the hybridoma-derived monoclonal antibody targeting IL13R*α*2 [20] is a promising candidate for GBM immunotherapy. The monoclonal antibody can be either delivered directly as antibody fragments with stabilizing agents, as it has been shown that targeting molecules, both antibodies as well as IL13R*α*2-targeted peptides has properties of homing to the tumor sites [24, 66]. Alternatively, delivery of a single chain variable fragment of the high-specificity monoclonal antibody can be achieved by expressing it as a CAR on engineered T cells, thereby increasing the efficiency of the immunotherapeutic procedure.

Dendritic cells pulsed with tumor-associated antigens are a successful GBM immunotherapeutic approach [67]. By loading the dendritic cells with anti-GBM information the immune system is retrained to identify the GBM tumor cells as a threat. GBM patients vaccinated with autologous dendritic cells pulsed with different glioma-associated tumor antigens, including IL13R*α*2, have shown significant prolongation of progression-free survival [28]. However, it has to be taken into account that none of the tumor-associated antigens used to pulse the dendritic cells are foreign antigens. This significantly blunts the antitumor immune response due to increased tolerance to the self-antigens, thus limiting the effectiveness of this approach.

Although promising, none of the above findings are as startling as the potency of CAR T cell in other malignancies. While clinical experience with CAR T cells for GBM is limited, recent success in patients with hematological malignancies has highlighted their antitumor potency [10, 75–77]. CARs combine the antigen-binding and lytic properties of monoclonal antibody or a simple ligand-receptor binding properties along with the self-renewing capacities of T cells [32, 34, 78, 79]. CAR T cells act on tumor cells in a MHC-independent fashion and therefore remain unaffected by the major mechanisms by which tumors evade the host immune system [33]. CARs can be designed to express either antibody to target peptide antigens on the tumor surface or ligands to target tumor-specific receptors (in this case IL13 muteins as ligands for IL13R*α*2 on GBMs). Because of this advantage CAR T cells have been developed against a plethora of GBM immunotherapy candidates, some of which have progressed onto clinical trials (Table 3). In preclinical tests, CAR T cells designed to target IL13R*α*2 produced copious amounts of immunostimulatory cytokines in presence of IL13R*α*2-expressing GBM tumor cell lines as well as patient tumor cells indicating the high specificity. In orthotopic xenograft glioma-bearing animal models, the IL13R*α*2-targeting CARs showed increased survival of treated animals when compared to untransduced T cells [22, 23]. IL13R*α*2-targeting CARs have also been successful against chemo- and radioresistant glioma-initiating cells which otherwise are the cause of recurrent GBM [41]. A recent bispecific CAR T cell developed to target both HER2 and IL13R*α*2 has shown promise to curb the immune-escape mechanism often exhibited by GBMs undergoing immunotherapy. Together, it appears that CAR T cells have immense potential as a candidate for targeted immunotherapy of GBM. However, questions, like delivery method of CAR T cells in hosts, survival in presence of lymphopenic chemotherapy drugs, and long-term host immune effect, remain unanswered, which may impose a limitation to this otherwise successful immunotherapeutic approach.

## Figures and Tables

**Table 1:** Chimeric antigen receptors against IL13R*α*2.

Chimeric antigen receptors	Mutation	Delivery	Clinical trials
IL13 zetakine [22]	Single (E13Y)	Plasmid	Yes [37, 38]
IL13 CAR [23]	Double (E13K and R109K)	Retrovirus	No
HER2.CD28 and IL13R*α*2.CD28 biCAR [39]	E13K	Retrovirus	No
Multiple [40]	E13K, E13Y, E13Y.K105R, E13K.K105R	Retrovirus	No

**Table 2:** IL13R*α*2-targeted immunotherapy.

Immunotherapy	References	Clinical trials
Monoclonal antibodies	[20, 24]	None
Pulsed dendritic cells	[21, 27]	NCT01280552 [28]
Chimeric antigen receptors	[22, 23, 39, 41]	NCT00730613 [37] NCT01082926 [38]

**Table 3:** Chimeric antigen receptors targeting GBM.

GBM targets	Preclinical studies	Clinical trials
EGFRvIII	[68–70]	NCT01454596 [71]
EphA2	[72]	None
HER2	[73]	NCT01109095 [74]
IL13R*α*2	[22, 23, 39–41]	NCT00730613 [37] NCT01082926 [38]

## References

[1] Rolle CE, Sengupta S, Lesniak MS (2010). Challenges in clinical design of immunotherapy trials for malignant glioma. *Neurosurgery Clinics of North America*.

[2] Ashby LS, Ryken TC (2006). Management of malignant glioma: steady progress with multimodal approaches. *Neurosurgical focus*.

[3] Stupp R, Hegi ME, Mason WP (2009). Effects of radiotherapy with concomitant and adjuvant temozolomide versus radiotherapy alone on survival in glioblastoma in a randomised phase III study: 5-year analysis of the EORTC-NCIC trial. *The Lancet Oncology*.

[4] Omuro A, DeAngelis LM (2013). Glioblastoma and other malignant gliomas: a clinical review. *JAMA*.

[5] Rolle CE, Sengupta S, Lesniak MS (2012). Mechanisms of immune evasion by gliomas. *Advances in Experimental Medicine and Biology*.

[6] Bao S, Wu Q, McLendon RE (2006). Glioma stem cells promote radioresistance by preferential activation of the DNA damage response. *Nature*.

[7] Frosina G (2009). DNA repair and resistance of gliomas to chemotherapy and radiotherapy. *Molecular Cancer Research*.

[8] Carpentier AF, Meng Y (2006). Recent advances in immunotherapy for human glioma. *Current Opinion in Oncology*.

[9] Wainwright DA, Nigam P, Thaci B, Dey M, Lesniak MS (2012). Recent developments on immunotherapy for brain cancer. *Expert Opinion on Emerging Drugs*.

[10] Porter DL, Levine BL, Kalos M, Bagg A, June CH (2011). Chimeric antigen receptor-modified T cells in chronic lymphoid leukemia. *The New England Journal of Medicine*.

[11] Thaci B, Brown CE, Binello E, Werbaneth K, Sampath P, Sengupta S (2014). Significance of interleukin-13 receptor alpha 2-targeted glioblastoma therapy. *Neuro-Oncology*.

[12] Arima K, Sato K, Tanaka G (2005). Characterization of the interaction between interleukin-13 and interleukin-13 receptors. *Journal of Biological Chemistry*.

[13] Brown CE, Warden CD, Starr R (2013). Glioma IL13R*α*2 is associated with mesenchymal signature gene expression and poor patient prognosis. *PLoS ONE*.

[14] Debinski W, Gibo DM, Hulet SW, Connor JR, Gillespie GY (1999). Receptor for interleukin 13 is a marker and therapeutic target for human high-grade gliomas. *Clinical Cancer Research*.

[15] Debinski W, Obiri NI, Powers SK, Pastan I, Puri RK (1995). Human glioma cells overexpress receptors for interleukin 13 and are extremely sensitive to a novel chimeric protein composed of interleukin 13 and Pseudomonas exotoxin. *Clinical Cancer Research*.

[16] Madhankumar AB, Slagle-Webb B, Mintz A, Sheehan JM, Connor JR (2006). Interleukin-13 receptor-targeted nanovesicles are a potential therapy for glioblastoma multiforme. *Molecular Cancer Therapeutics*.

[17] Madhankumar AB, Slagle-Webb B, Wang X (2009). Efficacy of interleukin-13 receptor-targeted liposomal doxorubicin in the intracranial brain tumor model. *Molecular Cancer Therapeutics*.

[18] Candolfi M, Xiong W, Yagiz K (2010). Gene therapy-mediated delivery of targeted cytotoxins for glioma therapeutics. *Proceedings of the National Academy of Sciences of the United States of America*.

[19] Ulasov IV, Tyler MA, Han Y, Glasgow JN, Lesniak MS (2007). Novel recombinant adenoviral vector that targets the interleukin-13 receptor *α*2 chain permits effective gene transfer to malignant glioma. *Human Gene Therapy*.

[20] Balyasnikova IV, Wainwright DA, Solomaha E (2012). Characterization and immunotherapeutic implications for a novel antibody targeting interleukin (IL)-13 Receptor *α*2. *The Journal of Biological Chemistry*.

[21] Okada H, Kalinski P, Ueda R (2011). Induction of CD8^+^ T-cell responses against novel glioma-associated antigen peptides and clinical activity by vaccinations with *α*-type 1 polarized dendritic cells and polyinosinic-polycytidylic acid stabilized by lysine and carboxymethylcellulose in patients with recurrent malignant glioma. *Journal of Clinical Oncology*.

[22] Kahlon KS, Brown C, Cooper LJN, Raubitschek A, Forman SJ, Jensen MC (2004). Specific recognition and killing of glioblastoma multiforme by interleukin 13-zetakine redirected cytolytic T cells. *Cancer Research*.

[23] Kong S, Sengupta S, Tyler B (2012). Suppression of human glioma xenografts with second-generation IL13R-specific chimeric antigen receptor-modified T cells. *Clinical Cancer Research*.

[24] Kioi M, Seetharam S, Puri RK (2008). Targeting IL-13RA2-positive cancer with a novel recombinant immunotoxin composed of a single-chain antibody and mutated Pseudomonas exotoxin. *Molecular Cancer Therapeutics*.

[25] Fadul CE, Fisher JL, Hampton TH (2011). Immune response in patients with newly diagnosed glioblastoma multiforme treated with intranodal autologous tumor lysate-dendritic cell vaccination after radiation chemotherapy. *Journal of Immunotherapy*.

[26] Shimato S, Natsume A, Wakabayashi T (2008). Identification of a human leukocyte antigen-A24-restricted T-cell epitope derived from interleukin-13 receptor *α*2 chain, a glioma-associated antigen: laboratory investigation. *Journal of Neurosurgery*.

[27] Iwami K, Shimato S, Ohno M (2012). Peptide-pulsed dendritic cell vaccination targeting interleukin-13 receptor alpha2 chain in recurrent malignant glioma patients with HLA-A∗24/A∗02 allele. *Cytotherapy*.

[28] A Study of ICT-107 Immunotherapy in Glioblastoma Multiforme (GBM). http://clinicaltrials.gov/show/NCT01280552.

[29] Saikali S, Avril T, Collet B (2007). Expression of nine tumour antigens in a series of human glioblastoma multiforme: interest of EGFRvIII, IL-13R*α*2, gp100 and TRP-2 for immunotherapy. *Journal of Neuro-Oncology*.

[30] Okada H, Butterfield L, Hamilton R (2013). Robust inductions of type-1 CD8+ T-cell responses in WHO grade II low-grade glioma patients receiving peptide-based vaccines in combination with poly-ICLC. *Neuro-Oncology*.

[31] Ma Q, Gonzalo-Daganzo RM, Junghans RP (2002). Genetically engineered T cells as adoptive immunotherapy of cancer. *Cancer Chemotherapy and Biological Response Modifiers*.

[32] Jensen MC, Riddell SR (2014). Design and implementation of adoptive therapy with chimeric antigen receptor-modified T cells. *Immunological Reviews*.

[33] Krebs S, Rodriguez-Cruz TG, Derenzo C (2013). Genetically modified T cells to target glioblastoma. *Frontiers in Oncology*.

[34] Vonderheide RH, June CH (2014). Engineering T cells for cancer: our synthetic future. *Immunological Reviews*.

[35] Park TS, Rosenberg SA, Morgan RA (2011). Treating cancer with genetically engineered T cells. *Trends in Biotechnology*.

[36] Emtage PCR, Lo ASY, Gomes EM, Liu DL, Gonzalo-Daganzo RM, Junghans RP (2008). Second-generation anti-carcinoembryonic antigen designer T cells resist activation-induced cell death, proliferate on tumor contact, secrete cytokines, and exhibit superior antitumor activity in vivo: a preclinical evaluation. *Clinical Cancer Research*.

[37] Cellular Adoptive Immunotherapy Using Genetically Modified T-Lymphocytes in Treating Patients With Recurrent or Refractory High-Grade Malignant Glioma. http://clinicaltrials.gov/ct2/show/NCT00730613.

[38] Phase I Study of Cellular Immunotherapy for Recurrent/Refractory Malignant Glioma Using Intratumoral Infusions of GRm13Z40-2, An Allogeneic CD8+ Cytolitic T-Cell Line Genetically Modified to Express the IL 13-Zetakine and HyTK and to be Resistant to Glucocorticoids, in Combination With Interleukin-2. http://www.clinicaltrials.gov/ct2/show/NCT01082926.

[39] Hegde M, Corder A, Chow KK (2013). Combinational targeting offsets antigen escape and enhances effector functions of adoptively transferred T cells in glioblastoma. *Molecular Therapy*.

[40] Krebs S, Chow KK, Yi Z (2014). T cells redirected to interleukin-13Ralpha2 with interleukin-13 mutein-chimeric antigen receptors have anti-glioma activity but also recognize interleukin-13Ralpha1. *Cytotherapy*.

[41] Brown CE, Starr R, Aguilar B (2012). Stem-like tumor-initiating cells isolated from IL13R*α*2 expressing gliomas are targeted and killed by IL13-zetakine-redirected T cells. *Clinical Cancer Research*.

[42] Yaghoubi SS, Jensen MC, Satyamurthy N (2009). Noninvasive detection of therapeutic cytolytic T cells with 18 F-FHBG PET in a patient with glioma. *Nature Clinical Practice Oncology*.

[43] Jian GZ, Eguchi J, Kruse CA (2007). Antigenic profiling of glioma cells to generate allogeneic vaccines or dendritic cell-based therapeutics. *Clinical Cancer Research*.

[44] Liang Y, Diehn M, Watson N (2005). Gene expression profiling reveals molecularly and clinically distinct subtypes of glioblastoma multiforme. *Proceedings of the National Academy of Sciences of the United States of America*.

[45] Cowan JA, Thompson BG, Doherty GM (2010). Chapter 36. Neurosurgery. *CURRENT Diagnosis & Treatment: Surgery, 13e*.

[46] Fan Z, Sehm T, Rauh M (2014). Dexamethasone alleviates tumor-associated brain damage and angiogenesis. *PLoS ONE*.

[47] Bavaresco L, Bernardi A, Braganhol E, Wink MR, Battastini AMO (2007). Dexamethasone inhibits proliferation and stimulates ecto-5
′-nucleotidase/CD73 activity in C6 rat glioma cell line. *Journal of Neuro-Oncology*.

[48] Higgins SC, Pelkington GJ (2010). The in vitro effects of tricyclic drugs and dexamethasone on cellular respiration of malignant glioma. *Anticancer Research*.

[49] Nafe LA, Dodam JR, Reinero CR (2014). In-vitro immunosuppression of canine T-lymphocyte-specific proliferation with dexamethasone, cyclosporine, and the active metabolites of azathioprine and leflunomide in a flow-cytometric assay. *Canadian Journal of Veterinary Research*.

[50] Löwenberg M, Verhaar AP, van den Brink GR, Hommes DW (2007). Glucocorticoid signaling: a nongenomic mechanism for T-cell immunosuppression. *Trends in Molecular Medicine*.

[51] Liao J, Wang X, Bi Y (2014). Dexamethasone potentiates myeloid-derived suppressor cell function in prolonging allograft survival through nitric oxide. *Journal of Leukocyte Biology*.

[52] Kumaresan PR, Manuri PR, Albert ND (2014). Bioengineering T cells to target carbohydrate to treat opportunistic fungal infection. *Proceedings of the National Academy of Sciences of the United States of America*.

[53] Mazur MD, Nguyen V, Fults DW (2012). Glioblastoma presenting with steroid-induced pseudoregression of contrast enhancement on magnetic resonance imaging. *Case Reports in Neurological Medicine*.

[54] FDA Approval for Temozolomide. http://www.cancer.gov/cancertopics/druginfo/fda-temozolomide.

[55] Sengupta S, Marrinan J, Frishman C, Sampath P (2012). Impact of temozolomide on immune response during malignant glioma chemotherapy. *Clinical and Developmental Immunology*.

[56] Brock CS, Newlands ES, Wedge SR (1998). Phase I trial of temozolomide using an extended continuous oral schedule. *Cancer Research*.

[57] Heimberger AB, Sun W, Hussain SF (2008). Immunological responses in a patient with glioblastoma multiforme treated with sequential courses of temozolomide and immunotherapy: case study. *Neuro-Oncology*.

[58] Gander M, Leyvraz S, Decosterd L (1999). Sequential administration of temozolomide and fotemustine: depletion of O6-alkyl guanine-DNA transferase in blood lymphocytes and in tumours. *Annals of Oncology*.

[59] Kizilarslanoglu MC, Aksoy S, Yildirim NO, Ararat E, Sahin I, Altundag K (2011). Temozolomide-related infections: review of the literature. *Journal of B.U.ON.*.

[60] Briegert M, Enk AH, Kaina B (2007). Change in expression of MGMT during maturation of human monocytes into dendritic cells. *DNA Repair*.

[61] Briegert M, Kaina B (2007). Human monocytes, but not dendritic cells derived from them, are defective in base excision repair and hypersensitive to methylating agents. *Cancer Research*.

[62] Sawai N, Zhou S, Vanin EF, Houghton P, Brent TP, Sorrentino BP (2001). Protection and in vivo selection of hematopoietic stem cells using temozolomide, O6-benzylguanine, and an alkyltransferase-expressing retroviral vector. *Molecular Therapy*.

[63] Debinski W, Gibo DM, Slagle B, Powers SK, Gillespie GY (1999). Receptor for interleukin 13 is abundantly and specifically over-expressed in patients with glioblastoma multiforme. *International Journal of Oncology*.

[64] Joshi BH, Plautz GE, Puri RK (2000). Interleukin-13 receptor chain: a novel tumor-associated transmembrane protein in primary explants of human malignant gliomas. *Cancer Research*.

[65] Kawakami M, Kawakami K, Takahashi S, Abe M, Puri RK (2004). Analysis of interleukin-13 receptor *α*2 expression in human pediatric brain tumors. *Cancer*.

[66] Pandya H, Gibo DM, Garg S, Kridel S, Debinski W (2012). An interleukin 13 receptor *α* 2-specific peptide homes to human Glioblastoma multiforme xenografts. *Neuro-Oncology*.

[67] Wheeler CJ, Black KL (2009). DCVax-brain and DC vaccines in the treatment of GBM. *Expert Opinion on Investigational Drugs*.

[68] Bullain SS, Sahin A, Szentirmai O (2009). Genetically engineered T cells to target EGFRvIII expressing glioblastoma. *Journal of Neuro-Oncology*.

[69] Choi BD, Suryadevara CM, Gedeon PC (2014). Intracerebral delivery of a third generation EGFRvIII-specific chimeric antigen receptor is efficacious against human glioma. *Journal of Clinical Neuroscience*.

[70] Morgan RA, Johnson LA, Davis JL (2012). Recognition of glioma stem cells by genetically modified T cells targeting EGFRvIII and development of adoptive cell therapy for glioma. *Human Gene Therapy*.

[71] White Blood Cells With Anti-EGFR-III for Malignant Gliomas. http://www.clinicaltrials.gov/ct2/show/NCT01454596.

[72] Chow KK, Naik S, Kakarla S (2013). T cells redirected to EphA2 for the immunotherapy of glioblastoma. *Molecular Therapy*.

[73] Ahmed N, Salsman VS, Kew Y (2010). HER2-specific T cells target primary glioblastoma stem cells and induce regression of autologous experimental tumors. *Clinical Cancer Research*.

[74] CMV-specific Cytotoxic T Lymphocytes Expressing CAR Targeting HER2 in Patients with GBM (HERT-GBM). http://www.clinicaltrials.gov/ct2/show/NCT01109095.

[75] Kalos M, Levine BL, Porter DL (2011). T cells with chimeric antigen receptors have potent antitumor effects and can establish memory in patients with advanced leukemia. *Science Translational Medicine*.

[76] Porter DL, Kalos M, Zheng Z, Levine B, June C (2011). Chimeric antigen receptor therapy for B-cell malignancies. *Journal of Cancer*.

[77] Grupp SA, Kalos M, Barrett D (2013). Chimeric antigen receptor-modified T cells for acute lymphoid leukemia. *New England Journal of Medicine*.

[78] Maher J (2012). Immunotherapy of malignant disease using chimeric antigen receptor engrafted T cells. *ISRN Oncology*.

[79] Sadelain M, Brentjens R, Rivière I (2013). The basic principles of chimeric antigen receptor design. *Cancer Discovery*.

